# Treatment for Severe Lupus Nephritis: A Cost-Effectiveness Analysis in China

**DOI:** 10.3389/fphar.2021.678301

**Published:** 2021-09-06

**Authors:** Zonglin Dai, Xi Zhang, Irene OL Wong, Eric HY Lau, Zhiming Lin

**Affiliations:** ^1^ School of Public Health, Li Ka Shing Faculty of Medicine, The University of Hong Kong, Pokfulam, Hong Kong, SAR China; ^2^ Division of Rheumatology, Third Affiliated Hospital of Sun Yat-sen University, Guangzhou, China; ^3^ Laboratory of Data Discovery for Health (D24H), Hong Kong Science and Technology Park, Hong Kong, Hong Kong, SAR China

**Keywords:** Cost-Effectiveness, severe lupus nephritis, Markov model, therapeutic strategies, clinical prognosis

## Abstract

**Background:** Lupus nephritis (LN) is the most common secondary glomerular diseases that will cause end-stage renal disease (ESRD) and renal-related death. The cost-effectiveness of various treatments for LN recommended by official guidelines has not been investigated in China. Our study is to evaluate clinical prognosis and cost-effectiveness of the current treatments for severe LN.

**Methods:** A Markov model was simulated for 1,000 LN patients of 30 years old, over a 3-years and 30-years lifetime horizon respectively. We assessed the cost-effectiveness of six therapeutic strategies from a societal perspective, with cyclophosphamide (CYC) or mycophenolate mofetil (MMF) induction therapy followed by CYC, MMF or azathioprine (AZA) maintenance therapy. Main outcomes included quality-adjusted life years (QALYs), incremental cost-effectiveness ratio (ICER) and clinical prognosis. One and three times gross domestic product (GDP) per capita were used as the willingness-to-pay (WTP) thresholds. We also carried out sensitivity analysis under a lifetime horizon.

**Results:** Compared with the baseline strategy of CYC induction and maintenance, for a 3-years horizon the most cost-effective strategy was CYC induction and AZA maintenance with $448 per QALY gained, followed by MMF induction and AZA maintenance which however was not cost-effective under the one times GDP per capita WTP threshold. For a lifetime horizon, CYC induction and AZA maintenance remained the most cost-effective strategy but MMF induction and maintenance became cost-effective under the one times GDP per capita WTP threshold and achieved a higher complete remission rate (57.2 versus 48.9%) and lower risks of ESRD (3.3 versus 5.8%) and all-cause mortality (36.0 versus 40.8%). The risk of developing ESRD during maintenance was the most influential parameter affecting ICER.

**Conclusions:** The strategy of CYC induction followed by AZA maintenance was the most cost-effective strategy in China for short-term treatment, while the strategy of MMF in both induction and maintenance became cost-effective and yielded more desirable clinical outcomes for lifetime treatment. The uncertainty analysis supported the need for monitoring the progression to ESRD.

## Introduction

Lupus nephritis (LN) is a common complication of systemic lupus erythematosus (SLE), a chronic inflammatory disease that may induce organ damage, typically the kidney. The frequency of developing LN from SLE varies worldwide, with 40–80% among Asians (Almaani et al., 2017). In China, LN has become the most common secondary glomerular diseases, accounting for over 50% of adults with SLE (Chinese Guidelines for Diagnostic and Treatment of Lupus Nephritis Writing Group, 2019). The standardized mortality ratio of LN patients was around six compared with the general population (Yap et al., 2012; Parikh et al., 2020). 10% of LN patients developed end-stage renal disease (ESRD) and mortality due to kidney disease was found to be 5–25% for patients with proliferative LN (Almaani et al., 2017; Parikh et al., 2020).

According to the latest treatment guidelines for LN from Chinese Medical Association (2019) (CMA) (Chinese Guidelines for Diagnostic and Treatment of Lupus Nephritis Writing Group, 2019), LN is classified into class I (minimal mesangial LN) to class VI (advanced sclerosing LN). The currently recommended first-line treatments include the basic treatment, hydroxychloroquine (HCQ) and glucocorticoids (GC), plus immunosuppressive (IS) therapy which mainly consists of cyclophosphamide (CYC), azathioprine (AZA) and mycophenolate mofetil (MMF). The American College of Rheumatology (2012) (ACR), the European League Against Rheumatism (2019) (EULAR) and GLADEL–PANLAR Latin American (2018) provided similar recommendations (Hahn et al., 2012; Pons-Estel et al., 2018; Fanouriakis et al., 2019). In general, patients diagnosed with class III (focal LN with less than 50% of glomeruli), class IV (diffuse LN with over 50% glomeruli) and class V LN (subepithelial immune deposits and membranous LN) in combination with class III or IV require more aggressive therapy, i.e. using IS drugs in additional to the basic treatment. Besides, class III and class IV patients account for 39–72% of all six pathologic types (Wang et al., 2018). Therefore, we focused on class III and IV LN patients, including class III/IV + V (hereafter referred as ‘severe LN patients’).

A two-phase paradigm was recommended for severe LN patients. In the first phase, patients received induction therapy to control the acute inflammatory injury of the kidney and to achieve complete remission (defined as urine protein-to-creatinine ratio <0.5 mg/ mmol with normal kidney function). The second phase is maintenance therapy, targeting at keeping complete remission and avoiding recurrence. It is common that LN patients received lifelong treatment which led to profound economic burden. In the United States (US), the annual medical expenditures of LN exceeded $46,000 (USD) per patient (Carls et al., 2009). Another report estimated that the total annual costs including outpatient, hospitalization, non-medical costs and indirect costs of SLE was over $6,000 (USD) in Shanghai, China (Zhang et al., 2017).

CYC and MMF are listed as the first-line drugs in induction therapy, and AZA and MMF are recommended in the maintenance phase. Although CYC was not recommended for the maintenance therapy by the guidelines, it is still used in China due its relatively low cost (Zhang et al., 2014).

To our knowledge, no integrated cost-effectiveness analysis has been carried out considering the two phases of induction and maintenance and their interplay. We designed this study with structured model and surveillance of ESRD and death to assess the cost-effectiveness of current LN treatment strategies in China.

## Materials and Methods

### Target Population and Therapeutic Strategies

Patients primarily diagnosed with class III, IV LN alone, and in combination with class V were targeted in our study. Milder class I and class II (mesangial proliferative LN) patients require basic treatment of HCQ and GC only without IS therapy. More severe class V (membranous LN) patients were treated based on their patient conditions and class VI (advanced sclerosing LN) patients require renal replacement therapy instead of using IS drugs. These patients were not considered in our study.

We referred to the CMA treatment guidelines for severe LN patients and current clinical practice in China in our study (Hahn et al., 2012; Pons-Estel et al., 2018; Chinese Guidelines for Diagnostic and Treatment of Lupus Nephritis Writing Group, 2019; Fanouriakis et al., 2019). We considered intravenous CYC which is the main route of administration for treating LN patients in China (Chinese Guidelines for Diagnostic and Treatment of Lupus Nephritis Writing Group, 2019). The recommended dosage is 0.5–1 g per month for CYC and 1.5–3 g/ day for MMF as the first-line IS drugs to treat LN during the 6-months induction therapy. Besides, patients treated with CYC or MMF also received HCQ (0.3–0.5 g/ day) and pulse GC (0.5–1 g/ day) for 3 days, followed by prednisone (0.5–1 mg/ kg/ day), reducing the dose gradually each month until 0.15 mg/ kg/ day. In the maintenance therapy, AZA and MMF were recommended as the first-line IS drugs with dosage 75–100 mg/ day and less than 2 g/ day respectively. The dosage of CYC in maintenance was 0.5–1.0 g/ m^2^ every 3 months (Contreras et al., 2004).

We assumed that the standard treatment (HCQ and GC) was used during the entire treatment. CYC or MMF was used for 6 months in initial induction therapy. If complete remission is achieved, patients will switch to maintenance therapy with CYC, AZA or MMF (Fanouriakis et al., 2019). Patients experiencing renal relapse after complete remission during the maintenance therapy were assumed to switch back to the same initial regimen as in the induction therapy. However, if complete remission is not achieved or only partial remission (defined as ≥50% reduction in proteinuria to subnephrotic levels) is achieved by one of the IS drugs (CYC or MMF) after 6 months, the same induction therapy is then extended for another 6 months. However, a switch to the other IS drug will be implemented if one-year induction fails under the same therapy (Chinese Guidelines for Diagnostic and Treatment of Lupus Nephritis Writing Group, 2019). According to ACR treatment guideline, rituximab (RTX) was typically used when both CYC and MMF fail, and we assumed that patients could not achieve complete remission if RTX also fail (Hahn et al., 2012).

Severe LN patients should always be treated with IS drugs in additional to standard treatment (Hahn et al., 2012). CYC has long been considered the gold standard in treating LN, with superior complete remission rate and cheapest direct treatment cost, hence it is still widely used in China to achieve renal remission and prevent renal flares, although it is associated with adverse events (AEs) including bone marrow suppression, infertility and malignancy. Hence, we defined baseline strategy (S1) as: initial induction with CYC followed by CYC maintenance (CYC→CYC). We considered other strategies which comprised of combinations between two drug choices (CYC and MMF) in the initial induction and three drug choices (CYC, AZA and MMF) in the maintenance phase (S2-S6) for comparison, namely MMF→CYC, CYC→AZA, MMF→AZA, CYC→MMF and MMF→MMF.

### The Analytic Model

#### Model Overview

A Markov model was designed to assess the cost-effectiveness of six therapeutic strategies for LN. The mean age of patients diagnosed with LN was around 30 years (Nossent and Koldingsnes, 2000; Dooley et al., 2011; Moroni et al., 2014; Wang et al., 2018), accordingly we simulated patients who met the treatment standards of severe LN from the same age. A lifetime horizon was modeled, given that continuous immunosuppressive therapy is needed to reduce SLE activity and ESRD and improve the quality of life for severe LN patients (Maroz and Segal, 2013). The life expectancy of Chinese LN patients was around 60 years, hence the lifetime horizon was set to be 30 years (Mok et al., 2013). The specific timeline of treatment has not been clearly stipulated by guidelines but it is recommended to receive at least 3-years maintenance. Therefore, we also evaluated cost-effectiveness over 3 years to assess the short-term outcomes. We adopted a societal perspective in the study and considered both direct and indirect costs. The transition period or cycle of the model was 6-months covering the induction period and evaluation of the therapy (Dooley et al., 2011). Hence in the model, we ran a total of 60 cycles to simulate the lifetime effect of disease progression with different treatment strategies. Main outcomes from the model included the cost of each patient, cumulative quality-adjusted life years (QALYs), incremental cost per QALY and incremental cost-effectiveness ratio (ICER). ICER indicated additional costs per QALY gained compared with the previous least costly strategy. We also simulated the disease trends of ESRD and death.

#### Model Structure

**FIGURE 1:**
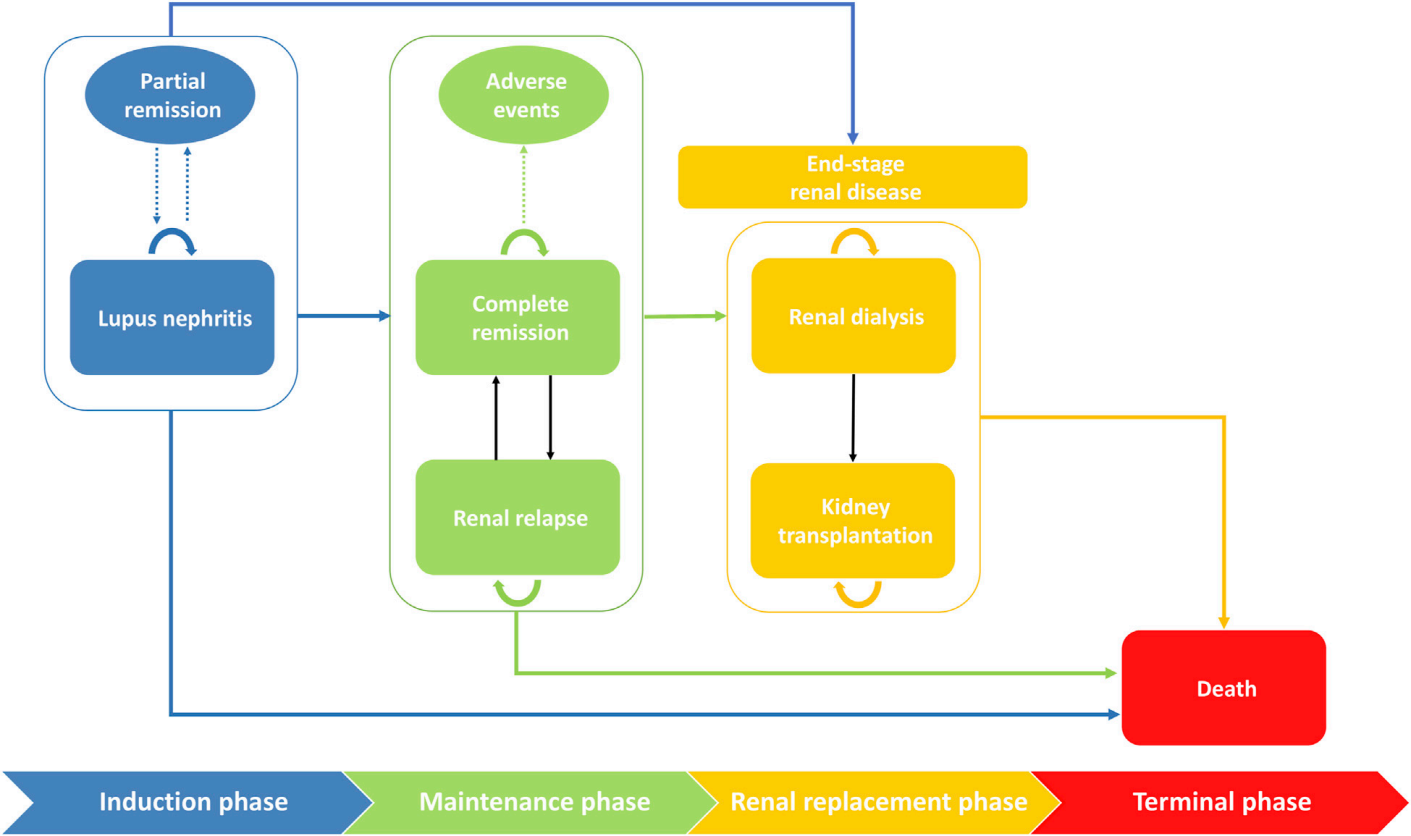
Markov structural model of health states with disease progression with 6-months cycles. The rounded rectangles represent health states and the ovals represent the potential outcomes in each phase. Transitions between phases or between states were indicated by solid arrows. The dotted arrows indicated potential outcomes. The initial phase is induction phase where patients start to receive therapy. After achieving complete remission, patients will progress to the maintenance therapy. Patients in both induction and maintenance phase may transit to renal replacement phase. All health states have a risk of death.

We considered four main phases of patient management, namely induction, maintenance, renal replacement, and terminal phase, with six health status including LN, complete remission, renal relapse, renal dialysis, kidney transplantation and death in our model (Figure 1). All severe LN patients received the induction therapy. Patients who achieved complete remission would progress to the maintenance phase but may still have a risk of relapse. A systematic review and meta-analysis found that renal dialysis was always considered as the initial renal replacement therapy, prior to transplantation (Swai et al., 2020). For simplicity, we assumed in our model that patients with ESRD received dialysis first, and may further require kidney transplantation if the patient’s condition deteriorated (Adler et al., 2006).

### Model Input

#### Transition Probability

We extracted the relevant transition probabilities between health status and their ranges for uncertainty analysis, based on an extensive literature review of primary studies and meta-analyses (Table 1). We extracted the drug efficiency data in induction phase, prioritizing head-to-head comparison studies. These parameters were converted for use in our model with a 6-months cycle (detailed description in the Supporting Material).

**TABLE 1:** Transition probabilities related to disease progress and different treatments^a^.

6-months transition	Estimate (%)	Range for sensitivity analysis^b^ (%)	References
Induction therapy with immunosuppressive drugs, from lupus nephritis			
To complete remission with CYC	40.84	21.74–66.67	Moroni et al. (2014)
To complete remission with MMF	31.37	25.00–54.00	Moroni et al. (2014)
To complete remission with RTX	45.78	14.20–72.70	Moroni et al. (2014)
To ESRD	0.80	0.71–0.84	Croca et al. (2011)
To lupus-related death	0.80	0.48–1.87	Croca et al. (2011)
To ESRD, when treatment failure^c^	2.48	0.81–8.00	Korbet et al. (2000)
To lupus-related death, when treatment failure	2.83	2.14–3.64	Korbet et al. (2000)
Maintenance therapy with immunosuppressive drugs, from complete remission			
To renal relapse with CYC	5.00	3.30–7.73	Nee et al. (2015); Pons-Estel et al. (2018)
To ESRD with CYC	0.45	0.23–0.96	Zhang et al. (2014)
To lupus-related death with CYC	1.84	0.33–13.08	Nee et al. (2015); Tunnicliffe et al. (2018)
To renal relapse with AZA	3.64	2.34–5.87	Nee et al. (2015)
To ESRD with AZA	0.30	0.06–1.60	Nee et al. (2015)
To lupus-related death with AZA	0.25	0.04–1.57	Nee et al. (2015)
To renal relapse with MMF	1.85	1.22–2.86	Nee et al. (2015)
To ESRD with MMF	0.12	0.02–0.63	Nee et al. (2015)
To lupus-related death with MMF	0.43	0.07–2.85	Nee et al. (2015)
Renal replacement therapy			
Transition probability to KT after receiving renal dialysis	0.85	0.36–2.01	Yikui et al. (2015); Wang et al. (2019)
Transition probability to death after receiving renal dialysis	4.29	1.00–7.47	Wu et al. (2014); Tsai et al. (2019)
Transition probability to death after receiving KT	0.29	0.19–0.37	Wu et al. (2014); Tsai et al. (2019)

AZA, azathioprine; CYC, cyclophosphamide; ESRD, end-stage renal disease; KT, kidney transplantation; MMF, mycophenolate mofetil; RTX, rituximab.

a The estimates and validations regarding the treatment with immunosuppressive drugs were referenced to Bernardo et al., 2018 (Pons-Estel et al., 2018) and (Tunnicliffe et al., 2018).

b Ranges for the uncertainty analysis were either obtained from the range of estimates in systematic reviews, or from the 95% confidence intervals from a specific study.

c No complete remission after treatment with immunosuppressive drugs.

According to the guideline in from CMA, after the induction therapy, standard evaluation methods including clinical symptoms (kidney function) and indicators (urine protein-to-creatinine ratio) are used to measure the health status. Those who enter the maintenance therapy after reaching complete remission must have matched the above assessment (Chinese Guidelines for Diagnostic and Treatment of Lupus Nephritis Writing Group, 2019). In the simulation, we assumed that the effect of maintenance therapy was independent of previous states during induction (Chinese Guidelines for Diagnostic and Treatment of Lupus Nephritis Writing Group, 2019).

We searched PubMed, Web of Science, Google Scholar, China National Knowledge Infrastructure and Embase for articles with the keywords “lupus nephritis”, “induction”, “cyclophosphamide”, “mycophenolate mofetil” or/and “rituximab” in induction therapy. A similar search was carried out by changing the keyword “induction” to “maintenance”, and added “azathioprine” in maintenance therapy. In renal replacement phase, we used the keywords “end-stage renal disease”, “kidney transplantation”, “death” or/and “mortality” without restriction on language between 1980 and 2020. To obtain head-to-head transition probability, we reviewed the literature extensively. For instance, in induction therapy, transitions from LN to complete remission including CYC, MMF and RTX therapy were based on a clinical observational study, with converting the annual transition probability to 6-months cycle (Moroni et al., 2014).

#### Quality-Adjusted Life Years and Costs

We used QALYs as the utility measurement, calculated by multiplying the utility score by time spent in a state (Whitehead and Ali, 2010). The utility scores of various health status measured by EQ-5D index were extracted from the literature (Liem et al., 2008; Mohara et al., 2014). The state of death was assigned a utility score of 0, and the other states were assigned health utility score ranging from 0.56 to 0.94 (Supplementary Table S1). Due to the higher treatment costs for AEs and the significant infertility risk due to CYC, the estimated utility score for complete remission and renal relapse after being treated with CYC was lower than that treated with other IS drugs (McDermott and Powell, 1996; Nee et al., 2015; Jones et al., 2019).

In the model, we considered total costs associated with treatment and management of LN, including direct costs and indirect costs. Direct costs consisted of direct health care costs (drugs, treatment-related AEs, medical devices, diagnostic tests, laboratory tests, hospital admission fee, etc.) and direct non-medical costs (transportation, accommodation expenses and social service such as retraining). AEs included major and minor infections, pneumonia, gastrointestinal manifestation, and leucopenia induced by IS drugs, and diabetes, hypertension, fractures, and eye diseases induced by GC and HCQ. Risks of these AEs and the related costs were presented in (Supplementary Tables S2–4). Prices of GC and HCQ and IS drugs were obtained from Hospital Information System, the Third Affiliated Hospital of Sun Yat-sen University, a major medical center in China and the evaluation of the costs of AE was also based on the system by two physicians (XZ and ZL) (The Third Affiliated Hospital of Sun Yat-sen University, 2019). Indirect costs included productivity loss, calculated by multiplying gross value of daily average income per capita in China by days off work (Supplementary Table S5); (Jo, 2014). The major cost for living donor kidney transplantation was accrued shortly after the treatment, and the direct and indirect health costs dropped quickly afterwards (Supplementary Table 1).

Considering the similar utilities between hemodialysis (HD) and peritoneal dialysis (PD) and the more popular use of HD in China (Wang et al., 2006; Liem et al., 2008; Zhang and Zuo, 2016). we considered the costs of HD for patients with ESRD in the analysis. The costs of dialysis in renal replacement phase included medication, consultation, laboratory and radiological investigation, dialysis solution, machine depreciation and other costs. Similarly, we considered the costs of living donor kidney transplantation in the study which is most common in China. Costs of kidney transplantation also included surgical and nursing, laboratory and testing, immunosuppressive agents, accommodation and other costs (Xiaoming et al., 2012). All costs were converted to 2019 prices using the consumer price indices from 2003 to 2019, and from Chinese Yuan (CNY, ¥) to U.S. dollars (USD, $) using the exchange rate in 2019 (1 USD = 6.87 CNY) (National Bureau of Statistics of China, 2019). QALY and costs were discounted at a rate of 1.5% per 6-months cycle (3% per year) (Chhatwal et al., 2016). One times gross domestic product (GDP) per capita (¥70,892 or US$10,319, 2019) in mainland China was considered as the willingness-to-pay (WTP) threshold, which was considered highly cost–effective, and three times GDP per capita was also adopted as the threshold for being cost-effective (¥212,676 or US$30,957, 2019) (Marseille et al., 2015; National Bureau of Statistics of China, 2019).

### Uncertainty Analysis

We assessed the uncertainty of the estimates with deterministic sensitivity analysis (DSA) and probabilistic sensitivity analysis (PSA) over the lifetime horizon. Key parameters including the transition probability between health states, costs and utility of each health state and discount rate were varied sequentially in DSA (Table 1; Supplementary Table S1). Ranges for the uncertainty analysis were either obtained from the range of estimates in systematic reviews, or from the 95% confidence intervals from a specific study. The outcome was presented in tornado plots, showing the most influential parameters on model results. In PSA, pre-defined parameters were re-sampled from respective distributions with 1,000 simulated cohorts. Dirichlet, binomial, normal and gamma distributions were assumed in the transition probabilities between states, discount rate, utility and costs respectively (Chen et al., 2016).

## Results

Table 2 summarized cost effectiveness and outcomes of different LN treatment strategies by 3-years and lifetime horizon. The efficiency frontiers at lifetime horizons are presented in Supplementary Figure S1. For a 3-years horizon, treating LN patients with CYC induction therapy and AZA maintenance therapy (S3, $448 per QALY gained) was most cost-effective compared with the baseline strategy (S1: CYC→CYC), with 0.218 more QALYs (Table 2). S4 (MMF→AZA) was the next cost-effective strategy if one is willing to pay $22,262 more than S3 (CYC→AZA) at a WTP of three times GDP per capita (US$30,957) but was then dominated by the most effective strategy S6 (MMF→MMF) after 24 years (Supplementary Figure S2). The proportions of patients experiencing renal replacement was lower for strategies with MMF maintenance (S5 and S6), and there were lower complete remission rate and higher all-cause mortality for strategies with CYC maintenance (S1 and S2, Table 2). For a lifetime horizon, CYC induction and AZA maintenance therapy (S3) was also the most cost-effective strategy which was associated with 48.9, 5.8 and 40.8% complete remission rate, risk of renal replacement and all-cause mortality respectively. However, S6 (MMF→MMF) achieved the highest complete remission rate and the lowest risk of renal replacement and all-cause mortality, at 57.2, 3.3 and 36.0% respectively among all cost-effective strategies. Again, strategies with CYC maintenance (S1 and S2) had noticeably lower complete remission rate and higher mortality. Due to the small number of cases who developed ESRD (Supplementary Figure S3, costs were mainly driven drug costs (CYC, MMF or AZA). Cost associated with ESRD increased disproportionately in the long run but still much lower than the drug costs.

**TABLE 2:** Base-case cost-effectiveness outcomes of different strategies for LN treatment, and predicted cumulative incidence of complete remission, renal replacement and all-cause mortality.

Strategy	Cumulative costs (US$)	Cumulative QALYs	Incremental costs (US$)	Incremental QALYs	ICER (US$/QALY)	Complete remission^a^(%)	Renal replacement^b^ (%)	All-cause mortality^c^ (%)
3-years horizon								
S1: CYC→CYC	15,874	2.156	−	−	−	75.9	2.9	7.9
S3: CYC→AZA	15,972	2.374	98	0.218	448	82.5	2.5	3.4
S2: MMF→CYC	17,469	2.308	1,595	0.152	Dominated	75.4	3.0	7.6
S4: MMF→AZA	17,484	2.442	1,512	0.068	22,262	81.8	2.6	3.5
S5: CYC→MMF	17,594	2.384	1,622	0.010	Dominated	85.9	2.0	3.2
S6: MMF→MMF	18,897	2.452	1,413	0.010	136,075	85.3	2.1	3.4
Lifetime horizon								
S1: CYC→CYC	70,286	9.745	−	−	−	18.7	5.1	73.7
S2: MMF→CYC	76,480	10.702	6,194	0.957	Dominated	18.3	5.2	73.2
S3: CYC→AZA	82,540	14.287	12,254	4.542	2,698	48.9	5.8	40.8
S4: MMF→AZA	88,393	14.866	5,853	0.579	Dominated	47.1	6.0	41.4
S5: CYC→MMF	90,031	14.869	7,491	0.582	Dominated	58.5	3.2	35.5
S6: MMF→MMF	93,708	15.517	11,168	1.230	9,079	57.2	3.3	36.0

AZA, azathioprine; CYC, cyclophosphamide; ICER, incremental cost-effectiveness ratios; MMF, mycophenolate mofetil; QALYs, quality-adjusted life years.

a Chi-squared test for equality of proportions: *p* < 0.001 among strategies at 3-years horizon and *p* < 0.001 among strategies at lifetime horizon.

b Renal replacement included renal dialysis and kidney transplantation. Chi-squared test for equality of proportions: *p* = 0.658 among strategies at 3-years horizon and *p* = 0.005 among strategies at lifetime horizon.

c Chi-squared test: *p* < 0.001 among strategies at 3-years horizon and *p* < 0.001 among strategies at lifetime horizon.

### Sensitivity Analysis

**FIGURE 2:**
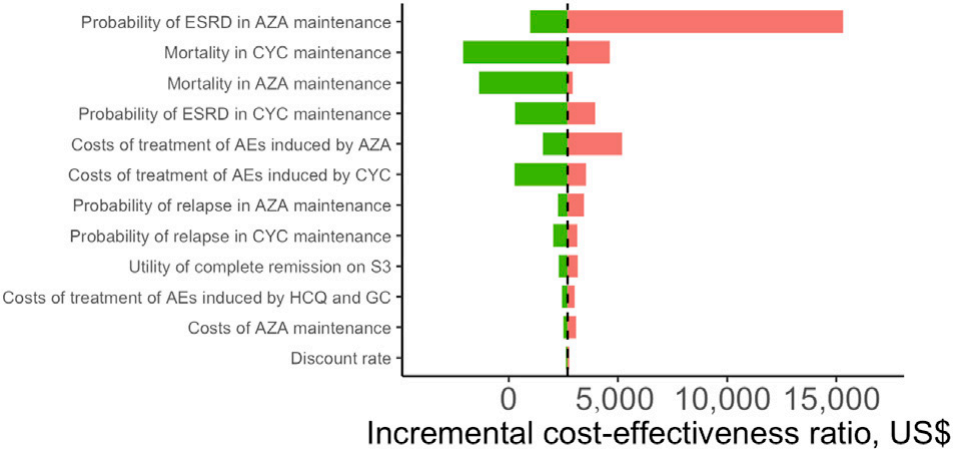
Tornado plot of deterministic sensitivity analysis for patients with lupus nephritis receiving the most cost-effective strategy (S3: CYC→AZA) compared with the baseline strategy (S1: CYC→CYC) over a lifetime horizon. The base-case result is presented by vertical dashed line. The length of the bars reflects the degree of parameters that influence quality-adjusted life years. Only the top 12 most influential parameters were presented. HCQ, hydroxychloroquine; GC, glucocorticoids; CYC, cyclophosphamide; AZA, azathioprine; ESRD, end-stage renal disease; AEs, adverse events.

**FIGURE 3:**
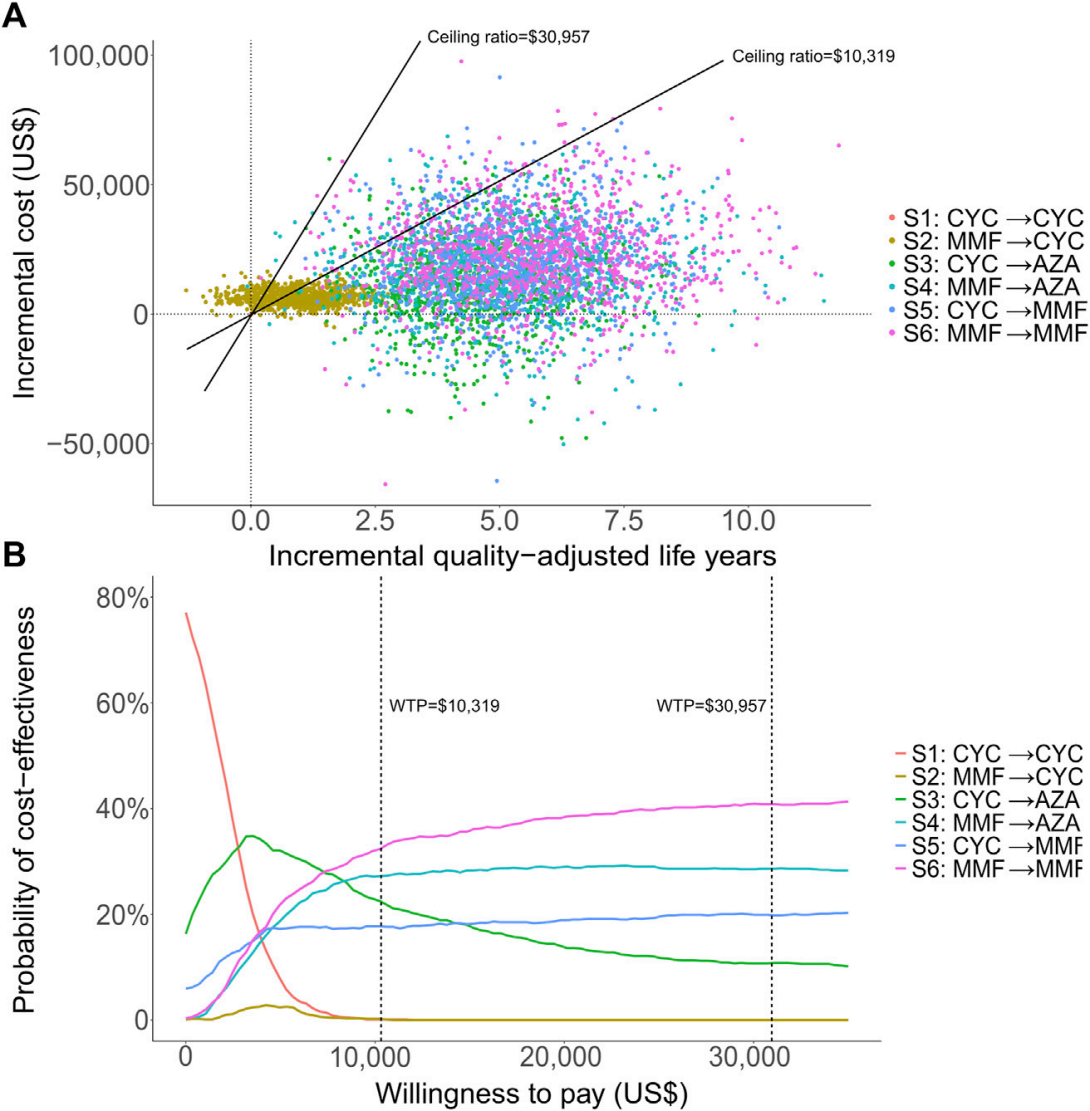
Probabilistic analytic results for the scenario over a lifetime horizon: the incremental cost-effectiveness differences simulated with 1,000 patients **(A)** and the cost-effectiveness acceptability curves of all strategies **(B)**. One-time and three-time gross domestic product per capita were used for the willingness to pay thresholds, at US$10,319 and US$30,957 respectively.

We conducted DSA for the most cost-effective strategy S3 (CYC→AZA) for a lifetime horizon (Figure 2). The most influential parameter that affected ICER was the risk of ESRD after complete remission during AZA maintenance therapy. Other influential parameters included the mortality risk associated with CYC and AZA maintenance therapy, probability of ESRD in CYC maintenance therapy, and costs of treatment-related AEs by AZA and CYC. In PSA we estimated that at the WTP of one times GDP per capita, most simulated cohorts treated with S6 (MMF in both treatment phases) over a lifetime horizon were under the ceiling ratio, and more than 99% of the cohorts were under the ceiling ratio for the three times GDP per capita WTP, meaning the cost-effectiveness. S6 had the highest acceptability of 34% among all strategies, followed by S4 (MMF→AZA) being cost-effective with 28% probability (Figure 3). The cost-effectiveness acceptability of S4 (MMF→AZA) and S5 (CYC→MMF) became stable whereas the probability of being cost-effective for S6 increased to 40% at the three times GDP per capita WTP threshold.

## Discussion

LN with subsequent development of ESRD has led to substantial mortality burden among patients with SLE (Almaani et al., 2017). Current LN therapies may cause complications such as infections, pneumonia, toxic retinopathy and diabetes which require further treatment and are associated with high financial burden. While various LN treatment options have been recommended, our study is first to evaluate the cost-effectiveness of these treatment strategies in an integrated framework considering induction, maintenance, renal replacement and terminal phases in China.

We found that the strategy of CYC induction followed by AZA maintenance therapy (S3) was the most cost-effective for both the 3-years and lifetime horizon. A study in Thailand found that the same strategy was the only cost-saving strategy (Mohara et al., 2014). The most effective strategy S6 (MMF → MMF) was not cost-effective at both WTP thresholds for a 3-years time horizon. However, it became affordable and most cost-effective for a lifetime horizon probably due to a lower relapse rate and risk of developing ESRD for MMF maintenance compared to AZA, which compensated the relatively high drug cost for MMF (Dooley et al., 2011). The cost-effective treatment identified in our study is likely applicable in the Asian settings. Although no similar cost-effectiveness study has been conducted in high income countries considering the induction and maintenance therapy together, several studies examined cost-effectiveness of the induction therapy and maintenance therapy separately. In the United Kingdom, MMF is costing US$3,100 less than CYC over the 24-week period in induction therapy based on the price in 2005 (Wilson et al., 2007). In the United States, MMF was found to be more cost-effective, with an ICER of $6,454/QALY compared to AZA in lifetime maintenance therapy (Nee et al., 2015). These studies showed that MMF was cost-effective for both induction and maintenance therapy, consistent with our results. Our study results are likely applicable to high and middle income countries.

Compared with other strategies, MMF maintenance was associated with the lowest risks of ESRD and death over 30 years (Table 2; Supplementary Figure S3). S5 (CYC→MMF) was also dominated by S6 (MMF→MMF) in our study. Further, S3 (CYC→AZA) and S4 (MMF→AZA) resulted in higher risk of ESRD than S6 (MMF→MMF) when longer course of treatment was adopted, with the discrepancy becoming more prominent starting from 5 years of treatment (Supplementary Figure S3). This was partly due to the higher renal relapse rate in AZA maintenance, which was also demonstrated by a previous systematic review (Tunnicliffe et al., 2018). We also showed that use of CYC in long-term maintenance therapy would result in lower complete remission rate, and higher risk of ESRD and death. A meta-analysis also found that using MMF was likely to produce better clinical outcome than CYC (Liu et al., 2012). In China, AZA treatment is subsidized, and the use of CYC maintenance for treating sever LN patients should be discouraged.

Disease progression rate to ESRD during AZA maintenance was found to be the most influential factor affecting the cost-effectiveness of S3 (CYC→AZA) (Figure 2). Clinically, identifying patients with higher risk of developing ESRD is important to reduce the risk of morbidity and mortality, which was also an important factor affecting cost-effectiveness. In the US, the incidence of LN-associated ESRD increased 5 times approximately from 1982 to 2004 (Maroz and Segal, 2013). A need for careful monitoring of severe LN patients for progression to ESRD is recommended, including continuous immunosuppressive medication, regular follow-up, histopathologic examination, assessment of renal indices and treatment response of LN during maintenance (Hahn et al., 2012).

As a validation of our model, considering the most cost-effective strategy (S3: CYC → AZA) and most effective strategy (S6: MMF → MMF), the risk of developing ESRD were 4.0 and 2.8% respectively by 6 years, consistent with a meta-analysis analyzing studies with follow-up from 3 to 6 years, in which the pooled risk of developing ESRD were 30 and 17 per 1,000 during maintenance therapy using AZA and MMF respectively (Tunnicliffe et al., 2018). The estimated risks of 10-years all-cause death were 11.8 and 10.7% under treatment with S3 (CYC → AZA) and S6 (MMF → MMF), similar to another epidemiological study showing that the patient survival in Asia (Hong Kong, Iran, and Japan) reached 92% with the effect of immunosuppressive therapies over the same time span (Yap and Chan, 2015).

Our study has several limitations. First, some parameters were not available from China. We used available data from other countries which were most relevant. Heath-related quality of life in LN patients was estimated from several other countries. Second, the dosage of CYC in maintenance therapy was not obtained from official guidelines as it is no longer recommended as first-line therapy (Hahn et al., 2012; Chinese Guidelines for Diagnostic and Treatment of Lupus Nephritis Writing Group, 2019). We assumed the decrease to half of the dosage in the maintenance phase was reflected in the drug price. Sensitivity analysis also showed that the cost of CYC had limited impact on the results. Third, some losses were difficult to measure in terms of exact costs, such as ovarian failure due to CYC where there is no effective way of prevention and treatment (McDermott and Powell, 1996). We also did not consider withdrawal of therapy due to more severe but rare AEs or other reasons. For example, monitoring of peripheral T lymphocytes are recommended when patients receive immunosuppression therapy (Houssiau et al., 2010). Dose reduction or even withdrawal of MMF should be considered if lymphocytes continue to decline, or CD4 + T cells are less than 200/ μL (Chinese Guidelines for Diagnostic and Treatment of Lupus Nephritis Writing Group, 2019). Lastly, though we have restricted our analysis to class III, IV, and III/IV + V LN patients and considered combination of drug options at the induction and maintenance therapy in each of which patients were more homogeneous in terms of disease severity, we could not rule out residual confounding by indication. It is also uncertain whether IS drug failure in the induction therapy would modify the efficacy of another IS drugs in the following induction or maintenance therapy, and we assumed efficacy of each therapy was independent.

In conclusion, our study demonstrated that for both a 3-years and lifetime horizon, the most cost-effective strategy for treating severe LN patients in China was CYC induction therapy, followed by AZA maintenance therapy at the three times GDP per capita WTP threshold. The strategy of using MMF in both induction and maintenance became cost-effective under the one times GDP per capita WTP threshold for a lifetime horizon, with clinical benefits of achieving the lowest ESRD and mortality among strategies considered. Monitoring of patients during maintenance for progression to ESRD is recommended.

## Data Availability

The original contributions presented in the study are included in the article/Supplementary Material, further inquiries can be directed to the corresponding authors.

## Glossary

LN: Lupus nephritis
SLE: Systemic lupus erythematosus
ESRD: End-stage renal disease
CMA: Chinese Medical Association
ACR: American College of Rheumatology
EULAR: European League Against Rheumatism
HCQ: Hydroxychloroquine
GC: Glucocorticoids
IS: Immunosuppressive
US: United States
USD ($): United States Dollar
CYC: Cyclophosphamide
AZA: Azathioprine
MMF: Mycophenolate mofetil
RTX: Rituximab
AE: Adverse event
QALY: Quality-adjusted life year
ICER: Incremental cost-effectiveness ratio
HD: Hemodialysis
PD: Peritoneal dialysis
CNY (¥): Chinese Yuan
GDP: Gross domestic product
WTP: Willingness-to-pay
DSA: Deterministic sensitivity analysis
PSA: Probabilistic sensitivity analysis
S1: Strategy 1
S2: Strategy 2
S3: Strategy 3
S4: Strategy 4
S5: Strategy 5
S6: Strategy 6
