# Supplementary figures and images for: Treatment for Severe Lupus Nephritis: A Cost-Effectiveness Analysis in China
Source: Front Pharmacol. 2021 Sep 6;12:678301. doi: 10.3389/fphar.2021.678301 (PMC8450585; doi:10.3389/fphar.2021.678301)

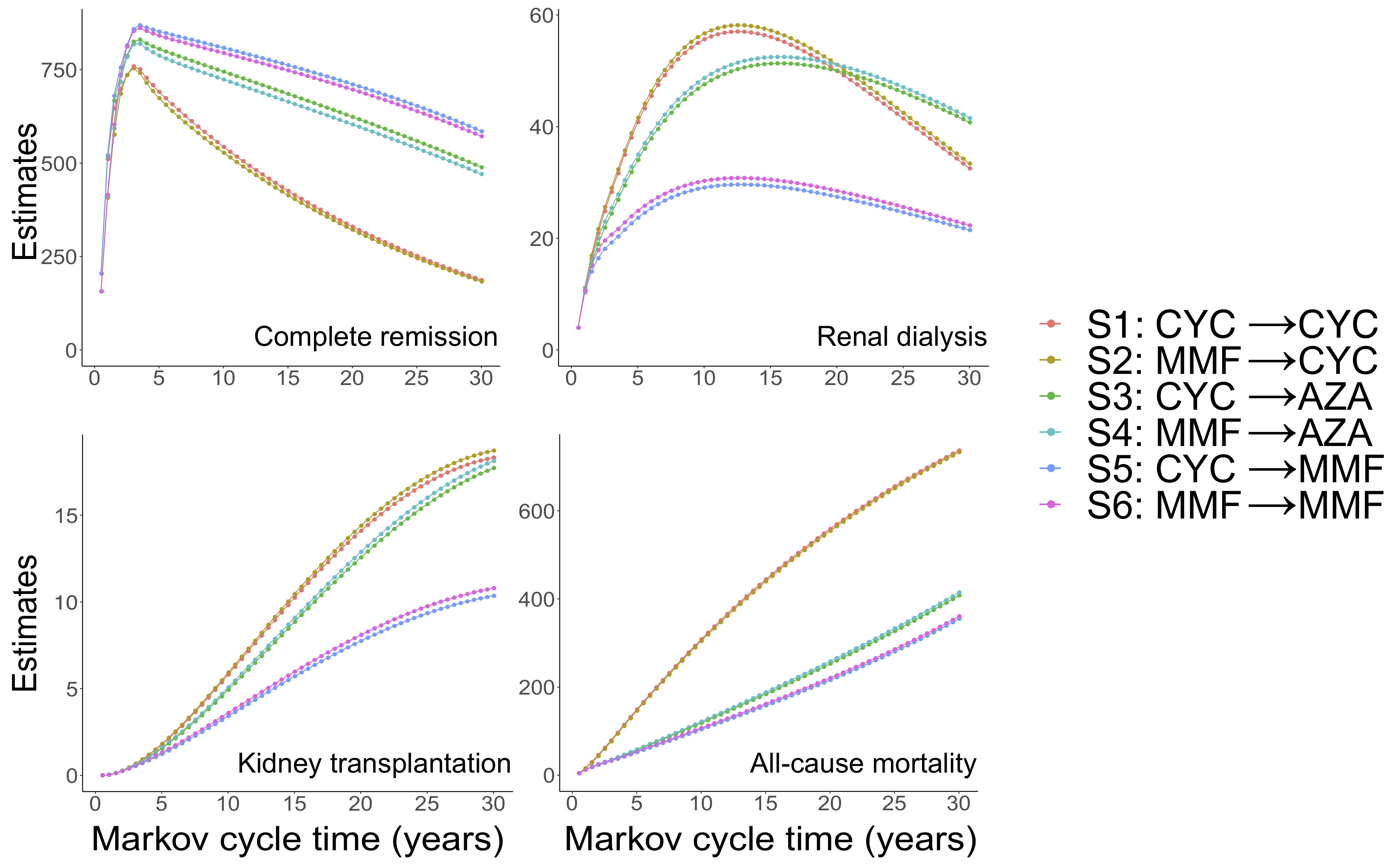

Supplement: Supplementary file 1 [file Image3.JPEG]

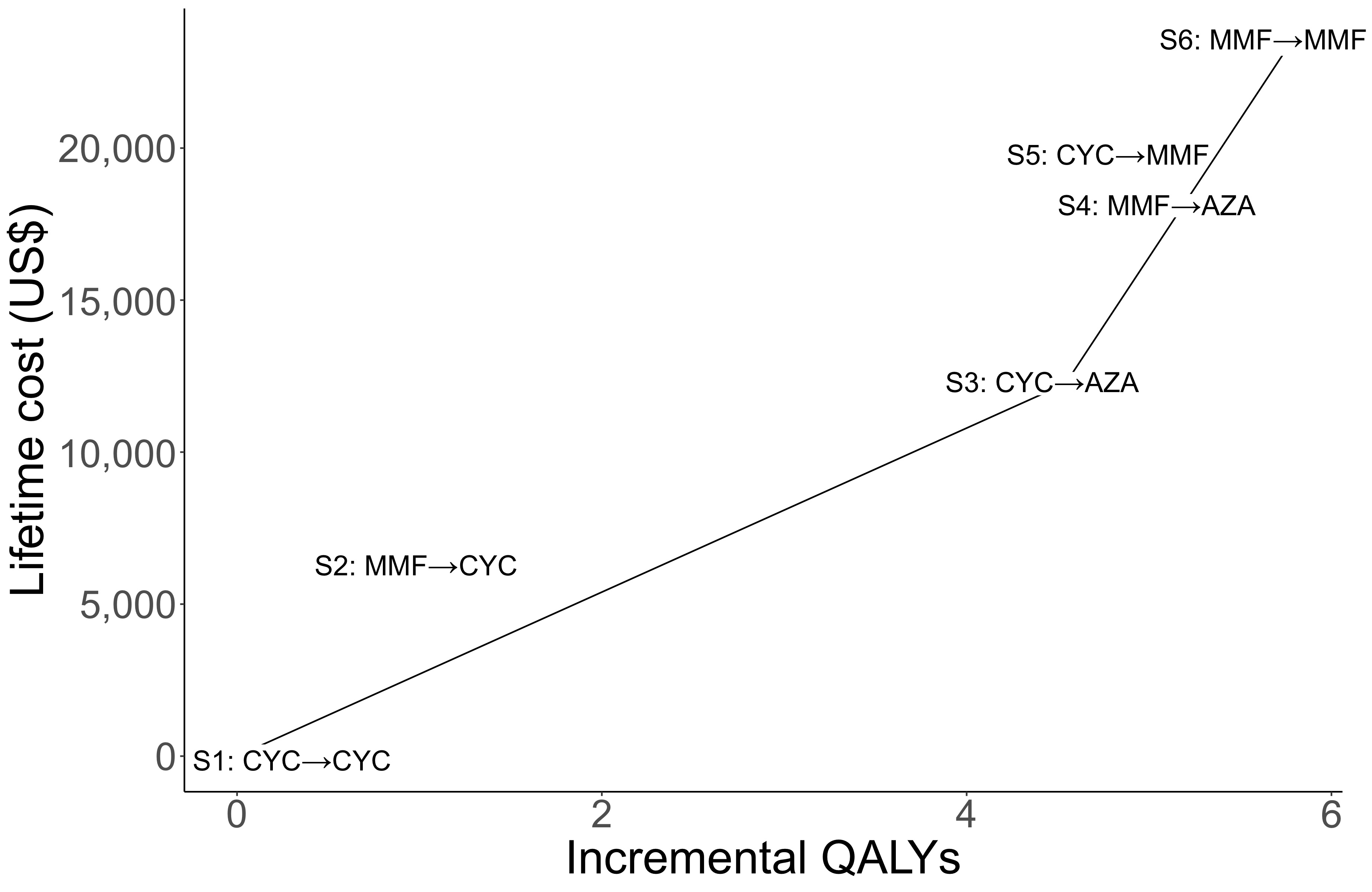

Supplement: Supplementary file 2 [file Image1.JPEG]

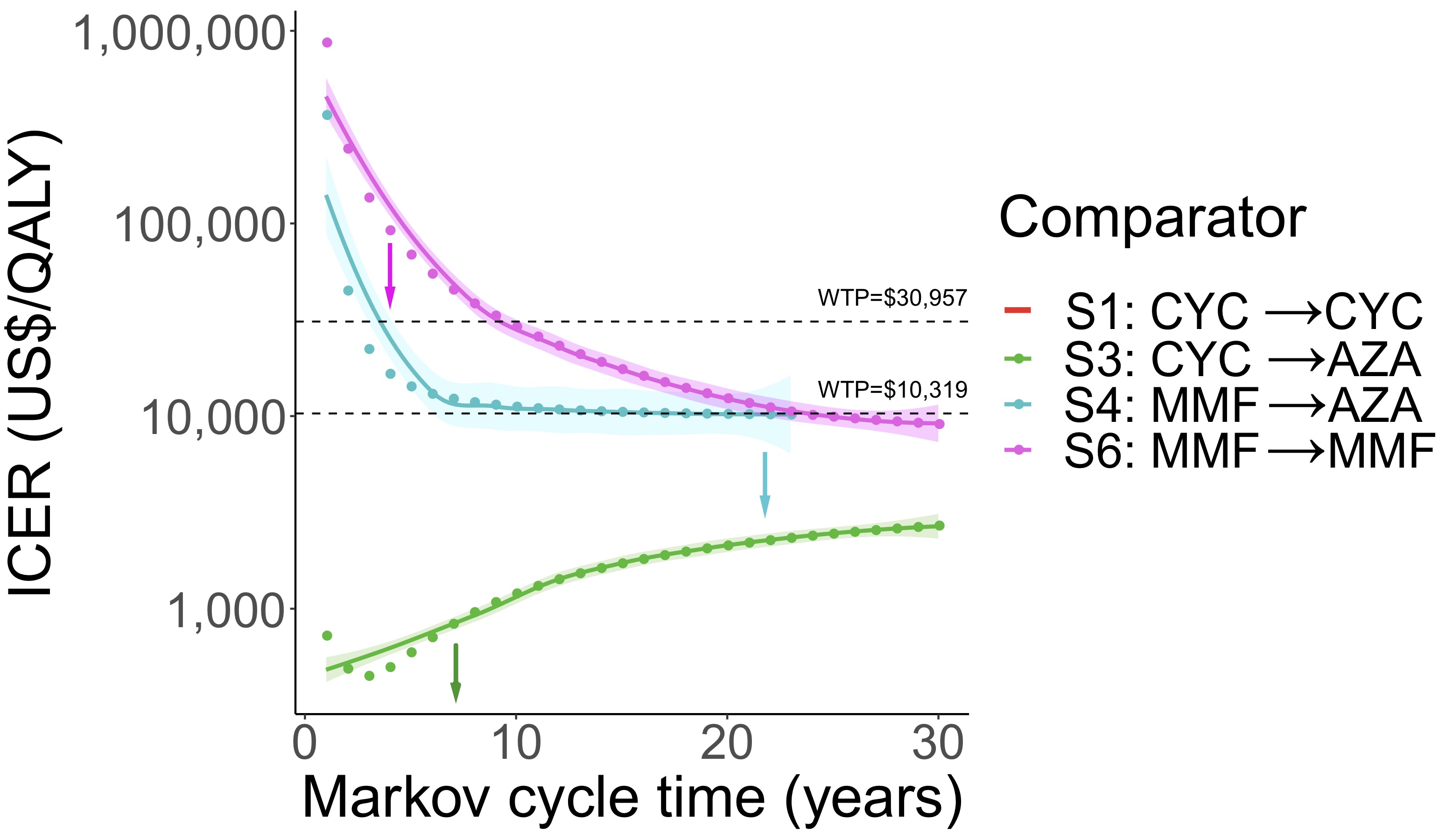

Supplement: Supplementary file 3 [file Image2.JPEG]
