# Supplementary material for: Treatment for Severe Lupus Nephritis: A Cost-Effectiveness Analysis in China
Source: Front Pharmacol. 2021 Sep 6;12:678301. doi: 10.3389/fphar.2021.678301 (PMC8450585; doi:10.3389/fphar.2021.678301)
Supplement: Supplementary file 4 [file DataSheet1.docx]

Supplementary Material

Table of Contents

[Description of the Markov model 2](#_Toc75357692)

[Supplementary Table 1 4](#_Toc75357693)

[Supplementary Table 2 7](#_Toc75357694)

[Supplementary Table 3 8](#_Toc75357695)

[Supplementary Table 4 9](#_Toc75357696)

[Supplementary Table 5 10](#_Toc75357697)

[Supplementary Figure 1 11](#_Toc75357698)

[Supplementary Figure 2 12](#_Toc75357699)

[Supplementary Figure 3 13](#_Toc75357700)

[References 14](#_Toc75357701)

Description of the Markov model

Four main phases of patient management, namely induction, maintenance, renal replacement and terminal phase were constructed, and six health states, namely lupus nephritis (LN), complete remission, renal relapse, renal dialysis, kidney transplantation and death were considered in our model (Figure 1). The arrows indicate the transition from one state to another. After constructing the six health states, we first assigned a hypothetical 1,000 cohorts to the initial state of LN. One cycle of the simulation was 6 months, which was the duration of one clinical treatment course. We ran the model with a short-term time horizon of 3 years (6 cycles) and a lifetime horizon of 30 years (60 cycles).

The total utility for each cycle was obtained by the sum of utility in all states for all 1,000 hypothetical LN patients. Cumulative utility was the sum of the cycle utility over 6 or 60 cycles. The cumulative cost was calculated similarly. Incremental cost-effectiveness ratio (ICER) was calculated by incremental costs between two competing strategies divided by incremental utility between two competing strategies. In our study, we used quality-adjusted life year (QALY) to measure utility, calculated by multiplying the utility score by time spent in a state.

We used age-specific all-cause and LN-related risk of death in our model. The probability of 6-month transition (p) between states was calculated based on the formula: ${p=1-(1-P)}^{1/2T}$, where P meant the rate of a disease under the certain time T. We also adjusted the annual discount rate r into 6-month cycle discount rate, by using ${r=(1+R)}^{1/2}-1$, where R represented the annual rate Chhatwal et al. (2016). In our study, we considered all-cause age-specific mortality to allow for age dependence. We used relative risk (RR) per cycle increase in the model, calculated by $RR=\sqrt[60]{UA/LA}$ where UA and LA were the mortality of the older persons (60 year-old) and the adults (30 year-old) respectively, based on National Bureau of Statistics of China National Bureau of Statistics of China). Risks of these AEs and the related costs were based on the following: $C=\sum_{k} c_{k}p_{k}$, where k represented the number of items, c was the estimated costs and p was the proportion of the AE.

The transition probability matrix between each state in the induction phase $\boldsymbol{P}_{induction}$ is given by:

$\boldsymbol{P}_{lnduction}=\left[ \begin{matrix} p_{LN\to LN} & p_{LN\to CR} & p_{LN\to ESRD} & p_{LN\to death} \\ 0 & p_{CR\to CR} & p_{CR\to ESRD} & p_{CR\to death} \\ 0 & 0 & p_{ESRD\to ESRD} & p_{ESRD\to death} \\ 0 & 0 & 0 & 1 \end{matrix} \right]$,

The transition probability matrix between each state in maintenance phase $\boldsymbol{P}_{maintenance}$ is given by:

$\boldsymbol{P}_{maintenance}=\left[ \begin{matrix} p_{CR\to CR} & p_{CR\to relapse} & p_{CR\to ESRD} & p_{CR\to death} \\ p_{relapse\to CR} & p_{relapse\to relapse} & p_{relapse\to ESRD} & p_{relapse\to death} \\ 0 & 0 & p_{ESRD\to ESRD} & p_{ESRD\to death} \\ 0 & 0 & 0 & 1 \end{matrix} \right]$,

The transition probability matrix between each state in renal replacement phase $\boldsymbol{P}_{renal replacement}$ is given by:

$\boldsymbol{P}_{renal replacement}=\left[ \begin{matrix} p_{RD\to RD} & p_{RD\to KT} & p_{RD\to death} \\ 0 & p_{KT\to KT} & p_{KT\to death} \\ 0 & 0 & 1 \end{matrix} \right]$.

Supplementary Table 1**.** 6-month base-case estimates of health-related states and disease burden.

| **Health states** | **Base-case utility**^*^ | | **Range for sensitivity analysis**^†^ | | **References** |
| --- | --- | --- | --- | --- | --- |
| Lupus nephritis | 0.76 | | 0.68-0.84 | | Mohara et al. (2014) |
| Complete remission, on treatment strategy: |  | |  | | Mohara et al. (2014); Nee et al. (2015) |
| S1: CYC→CYC | 0.80 | | 0.75-0.85 | |  |
| S2: MMF→CYC | 0.89 | | 0.84-0.95 | |  |
| S3: CYC→AZA | 0.89 | | 0.84-0.95 | |  |
| S4: MMF→AZA | 0.94 | | 0.88-0.99 | |  |
| S5: CYC→MMF | 0.89 | | 0.84-0.95 | |  |
| S6: MMF→MMF | 0.94 | | 0.88-0.99 | |  |
| Renal relapse, on treatment strategy: |  | |  | | Mohara et al. (2014); Nee et al. (2015) |
| S1: CYC→CYC | 0.62 | | 0.56-0.69 | |  |
| S2: MMF→CYC | 0.72 | | 0.65-0.80 | |  |
| S3: CYC→AZA | 0.72 | | 0.65-0.80 | |  |
| S4: MMF→AZA | 0.76 | | 0.68-0.84 | |  |
| S5: CYC→MMF | 0.72 | | 0.65-0.80 | |  |
| S6: MMF→MMF | 0.76 | | 0.68-0.84 | |  |
| Renal dialysis | 0.56 | | 0.49-0.62 | | Liem et al. (2008) |
| Kidney transplantation | 0.81 | | 0.72-0.90 | | Liem et al. (2008) |
| Death | 0 | | 0 | | Mohara et al. (2014) |
| **Disease cost burden** | **6-month base-case estimate** | | | | **References** |
|  | **CNY (¥)** | **Range for sensitivity analysis**^†^ **(¥)** | **USD ($)** | **Range for sensitivity analysis**^†^ **($)** |  |
| Direct health care costs |  |  |  |  | The Third Affiliated Hospital of Sun Yat-sen University (2019) |
| Drug costs^‡^ |  |  |  |  |  |
| HCQ for entire therapy | 1,871 | 1,403-2,339 | 272 | 204-340 |  |
| GC for induction therapy | 1,827 | 1,219-2,431 | 266 | 177-354 |  |
| GC for maintenance therapy | 417 | 278-556 | 61 | 41-81 |  |
| CYC induction therapy | 663 | 442-884 | 97 | 64-129 |  |
| MMF induction therapy | 10,100 | 7,575-15,150 | 1,470 | 1,103-2,205 |  |
| RTX induction therapy | 41,360 | 20,680-46,530 | 6,020 | 3,010-6,773 |  |
| CYC maintenance therapy | 324 | 221-442 | 47 | 32-64 |  |
| AZA maintenance therapy | 901 | 676-1,352 | 131 | 98-197 |  |
| MMF maintenance therapy | 5,050 | 3,788-7,575 | 735 | 551-1,103 |  |
| Costs of treatment-related AEs, caused by | |  |  |  | The Third Affiliated Hospital of Sun Yat-sen University (2019) |
| HCQ and GC | 2,835 | 1,826-4,090 | 413 | 266-595 |  |
| CYC | 2,782 | 1,377-6,808 | 405 | 200-991 |  |
| AZA | 2,483 | 1,166-5,371 | 361 | 170-782 |  |
| MMF | 1,904 | 935-4,662 | 277 | 136-679 |  |
| RTX | 2,774 | 1,373-6,153 | 404 | 200-896 |  |
| Other direct health care costs |  |  |  |  | Pan et al. (2012); Wang et al. (2006); Zhang et al. (2017) |
| Introduction therapy | 11,920 | 9,933-14,304 | 1,735 | 1,446-2,082 |  |
| Maintenance therapy | 1,265 | 650-2420 | 184 | 95-352 |  |
| Renal dialysis | 63,556 | 52,963-76,267 | 9,251 | 7,709-11,101 |  |
| Kidney transplantation^§^ | 229,091 | 138,762-319,422 | 33,347 | 20,198-46,495 |  |
| Post-kidney transplantation | 47,027 | 29,875-64,179 | 6,845 | 4,349-9,342 |  |
| Direct non-health care costs |  |  |  |  | Pan et al. (2012); Wang et al. (2006); Zhang et al. (2017) |
| Introduction therapy | 1,387 | 1,156-1,664 | 202 | 168-242 |  |
| Maintenance therapy | 550 | 245-1,100 | 80 | 36-160 |  |
| Renal dialysis | 4,592 | 3,827-5,510 | 668 | 557-802 |  |
| Kidney transplantation^§^ | 3,157 | 1,950-4,481 | 460 | 284-652 |  |
| Post-kidney transplantation | 1,085 | 670-1,540 | 158 | 98-224 |  |
| Indirect costs |  |  |  |  | National Bureau of Statistics of China (2019); Jo (2014) |
| Introduction therapy | 1,810 | 1,267-2,534 | 263 | 184-369 |  |
| Maintenance therapy | 543 | 181-905 | 79 | 26-132 |  |
| Renal dialysis | 11,584 | 9,593-13,575 | 1,686 | 1,396-1,976 |  |
| Kidney transplantation^§^ | 7,964 | 4,887-11,041 | 1,159 | 711-1,607 |  |
| Post-kidney transplantation | 905 | 181-1,629 | 132 | 26-237 |  |
| Abbreviations: AEs, adverse events; HCQ, hydroxychloroquine; GC, glucocorticoids; CYC, cyclophosphamide; MMF, mycophenolate mofetil; AZA, azathioprine; RTX, rituximab. | | | | | |
| ^*^ Estimates based on studies using EQ-5D-index.  ^†^ Ranges for utilities were obtained from the range of estimates in systematic reviews and ranges for costs were estimated from the minimum and maximum of drug dosage recommended by guidelines.  ^‡^ The cost of HCQ was calculated assuming a daily dosage of 0.4g over the entire therapy and the cost of GC was based on 0.75g pulse therapy daily for 3 days, followed by 0.75mg/kg per day and reduced to 0.15mg/kg per day at the sixth month. Input costs included CYC (0.75g/month), MMF (2g/day) and RTX (2g in the first month only) during induction. The cost of AZA was calculated assuming a dosage of 0.1g per day. The cost for MMF was halved during the maintenance phase according to the gradual reduction in the dosage and the cost of CYC was calculated with 0.75g/m^2^ every three month.  ^§^ Estimates from living donor kidney transplantation. | | | | | |

Supplementary Table 2. Estimated risks of common adverse events of patients during 6-month treatment of immunosuppressive (IS) drugs.

| **Adverse events** | **CYC (%)** | **AZA (%)** | **MMF (%)** | **RTX (%)** | **References** |
| --- | --- | --- | --- | --- | --- |
| Minor infections | 24.7 | 25.4 | 23.1 | 27.3 | Goswami et al. (2019); Rovin et al. (2012); Chan et al. (2005); Maneiro et al. (2014); Li et al. (2011); Appel et al. (2009); Dooley et al. (2011); Davies et al. (2013); Moroni et al. (2014) |
| Major infections | 0.7 | 1.1 | 0.6 | 1.4 |  |
| Pneumonia | 7.6 | 2.5 | 3.8 | 3.8 |  |
| Gastrointestinal manifestation | 0.5 | 0.5 | 0.9 | 0.5 |  |
| Leucopenia | 5.0 | 35.7 | 5.0 | 5.0 |  |

Supplementary Table 3. Risk of adverse events of patients treated with hydroxychloroquine and glucocorticoids.

| **Adverse events** | **6-month transition probability (%)** | **References** | |
| --- | --- | --- | --- |
| Toxic retinopathy | 0.6 | Melles and Marmor (2014) | |
| Diabetes | 1.8 | Ha et al. (2011) | |
| Hypertension | 5.5 | Costello et al. (2017) | |
| Fractures | 10.9 | Rossini et al. (2017) | |
| Eye diseases^*^ | 3.2 | Costello et al. (2017) | |
| Osteonecrosis | 1.9 | Nawata et al. (2018) | |
| ^*^ Eye diseases included cataract and glaucoma. | | | |

Supplementary Table 4. Costs related to adverse events over a 6-month treatment.

| **Adverse events** | **Costs*** | | | |
| --- | --- | --- | --- | --- |
|  | **CNY (¥)** | **Range for sensitivity analysis (¥)** | **USD ($)** | **Range for sensitivity analysis ($)** |
| Minor infections | 2,000 | 1,000-7,500 | 291 | 146-1,092 |
| Major infections | 100,000 | 50,000-150,000 | 14,556 | 7,278-21,834 |
| Pneumonia | 20,000 | 10,000-50,000 | 2,911 | 1,456-7,278 |
| Gastrointestinal manifestation | 3,500 | 1,000-6,000 | 509 | 146-873 |
| Leucopenia | 1,000 | 300-1,500 | 146 | 44-218 |
| Diabetes | 5,000 | 3,600-10,000 | 728 | 524-1,456 |
| Hypertension | 4,000 | 1,800-6,000 | 582 | 262-873 |
| Fractures | 15,000 | 10,000-20,000 | 2,183 | 1,456-2,911 |
| Eye disease^*^ | 10,000 | 6,000-20,000 | 1,456 | 873-2,911 |
| Osteonecrosis | 30,000 | 20,000-40,000 | 4,367 | 2,911-5,822 |

^*^ Based on the Hospital Information System, the Third Affiliated Hospital of Sun Yat-sen University.

^†^ Eye diseases included cataract and glaucoma.

Supplementary Table 5. Estimated duration of absence from work by health status.

| **Phase** | **Absence from work (days)** | **Range*** **(days)** | **References** |
| --- | --- | --- | --- |
| Induction therapy | 10 | 7-14 | The Third Affiliated Hospital of Sun Yat-sen University (2019); Pan et al. (2012); Zhang et al. (2017) |
| Maintenance therapy^†^ | 3 | 1-5 |  |
| Renal dialysis | 64 | 53-75 |  |
| Kidney transplantation | 44 | 27-61 |  |
| Post-kidney transplantation | 5 | 1-9 |  |
| ^*^ Ranges for absence from work were obtained from the estimate in systematic reviews. | | | |

^†^ The value and the range were extracted or derived from the Hospital Information System in the Third Affiliated Hospital of Sun Yat-sen University.

Supplementary Figure 1. Efficiency frontier for cost-effectiveness analysis at lifetime horizon. QALYs, quality-adjusted life years; CYC, cyclophosphamide; MMF, mycophenolate mofetil; AZA, azathioprine.

Supplementary Figure 2. Changes in incremental cost-effectiveness ratios (ICER) over simulated years. The smooth curves represent the variation of ICER as time increases, with the 95% confidence level shown as the shaded portions. The willingness to pay thresholds are set to one and three times gross domestic product per capita ($10,319 and $30,957 respectively). Arrows indicate comparison between another strategies. For example, in the 10-year cycle, S3 (CYC→AZA) was more cost-effective if one was willingness to pay US$1,199 more than S1(CYC→AZA). S4 (MMF→AZA) was more cost-effective if one was willingness to pay US$11,179 than S3. Furthermore, S6 (MMF→MMF) was more cost-effective if one was willingness to pay US$29,125 more than S4 which was dominated by S6 after 23 years. The willingness to pay threshold is one times and three times gross domestic product per capita of US$10,319, US$30,957 respectively.

Supplementary Figure 3. Simulated number of complete remissions, renal dialysis kidney transplantation and all-cause mortality per 1,000 patients over simulated years.

# References

Appel, G.B., Contreras, G., Dooley, M.A., Ginzler, E.M., Isenberg, D., Jayne, D., et al. (2009). Mycophenolate mofetil versus cyclophosphamide for induction treatment of lupus nephritis. *J Am Soc Nephrol* 20(5)**,** 1103-1112. doi: 10.1681/asn.2008101028.

Chan, T.M., Tse, K.C., Tang, C.S.O., Mok, M.Y., and Li, F.K. (2005). Long-term study of mycophenolate mofetil as continuous induction and maintenance treatment for diffuse proliferative lupus nephritis. *J Am Soc Nephrol* 16(4)**,** 1076-1084. doi: 10.1681/asn.2004080686.

Chhatwal, J., Jayasuriya, S., and Elbasha, E.H. (2016). Changing Cycle Lengths in State-Transition Models: Challenges and Solutions. *Medical Decision Making An International Journal of the Society for Medical Decision Making* 36(8)**,** 952.

Costello, R., Patel, R., Humphreys, J., McBeth, J., and Dixon, W.G. (2017). Patient perceptions of glucocorticoid side effects: a cross-sectional survey of users in an online health community. *BMJ Open* 7(4)**,** e014603. doi: 10.1136/bmjopen-2016-014603.

Davies, R.J., Sangle, S.R., Jordan, N.P., Aslam, L., Lewis, M.J., Wedgwood, R., et al. (2013). Rituximab in the treatment of resistant lupus nephritis: therapy failure in rapidly progressive crescentic lupus nephritis. *Lupus* 22(6)**,** 574-582. doi: 10.1177/0961203313483376.

Dooley, M.A., Jayne, D., Ginzler, E.M., Isenberg, D., Olsen, N.J., Wofsy, D., et al. (2011). Mycophenolate versus azathioprine as maintenance therapy for lupus nephritis. *N Engl J Med* 365(20)**,** 1886-1895. doi: 10.1056/nejmoa1014460.

Goswami, R.P., Sircar, G., Sit, H., Ghosh, A., and Ghosh, P. (2019). Cyclophosphamide versus mycophenolate versus rituximab in lupus nephritis remission induction: a historical head-to-head comparative study. *J Clin Rheumatol* 25(1)**,** 28-35. doi: 10.1097/RHU.0000000000000760.

Ha, Y.J., Lee, K.H., Jung, S.J., Lee, S.W., Lee, S.K., and Park, Y.B. (2011). Glucocorticoid-induced diabetes mellitus in patients with systemic lupus erythematosus treated with high-dose glucocorticoid therapy. *Lupus* 20(10)**,** 1027-1034. doi: 10.1177/0961203311402246.

Jo, C. (2014). Cost-of-illness studies: concepts, scopes, and methods. *Clin Mol Hepatol* 20(4)**,** 327-337. doi: 10.3350/cmh.2014.20.4.327.

Li, X., Ren, H., Zhang, Q., Zhang, W., Wu, X., Xu, Y., et al. (2011). Mycophenolate mofetil or tacrolimus compared with intravenous cyclophosphamide in the induction treatment for active lupus nephritis. *Nephrol Dial Transplant* 27(4)**,** 1467-1472. doi: 10.1093/ndt/gfr484.

Liem, Y.S., Bosch, J.L., and Myriam Hunink, M.G. (2008). Preference-based quality of life of patients on renal replacement therapy: a systematic review and meta-analysis. *Value Health* 11(4)**,** 733-741. doi: 10.1111/j.1524-4733.2007.00308.x.

Maneiro, J.R., Lopez-Canoa, N., Salgado, E., and Gomez-Reino, J.J. (2014). Maintenance therapy of lupus nephritis with mycophenolate or azathioprine: systematic review and meta-analysis. *Rheumatology (Oxford)* 53(5)**,** 834-838. doi: 10.1093/rheumatology/ket429.

Melles, R.B., and Marmor, M.F. (2014). The risk of toxic retinopathy in patients on long-term hydroxychloroquine therapy. *JAMA Ophthalmol* 132(12)**,** 1453-1460. doi: 10.1001/jamaophthalmol.2014.3459.

Mohara, A., Pérez Velasco, R., Praditsitthikorn, N., Avihingsanon, Y., and Teerawattananon, Y. (2014). A cost-utility analysis of alternative drug regimens for newly diagnosed severe lupus nephritis patients in Thailand. *Rheumatology (Oxford)* 53(1)**,** 138-144. doi: 10.1093/rheumatology/ket304.

Moroni, G., Raffiotta, F., Trezzi, B., Giglio, E., Mezzina, N., Del Papa, N., et al. (2014). Rituximab vs mycophenolate and vs cyclophosphamide pulses for induction therapy of active lupus nephritis: a clinical observational study. *Rheumatology (Oxford)* 53(9)**,** 1570-1577. doi: 10.1093/rheumatology/ket462.

National Bureau of Statistics of China Tabulation on the 2010 Population Census of the People's Republic of China. Available from: <http://www.stats.gov.cn/tjsj/pcsj/rkpc/6rp/indexch.htm>.

National Bureau of Statistics of China (2019). Annual data. Available from: <http://data.stats.gov.cn/>.

Nawata, K., Nakamura, J., Ikeda, K., Furuta, S., Nakajima, H., Ohtori, S., et al. (2018). Transitional changes in the incidence of osteonecrosis in systemic lupus erythematosus patients: focus on immunosuppressant agents and glucocorticoids. *Rheumatology (Oxford)* 57(5)**,** 844-849. doi: 10.1093/rheumatology/key009.

Nee, R., Rivera, I., Little, D.J., Yuan, C.M., and Abbott, K.C. (2015). Cost-utility analysis of mycophenolate mofetil versus azathioprine based regimens for maintenance therapy of proliferative lupus nephritis. *Int J Nephrol* 2015**,** 917567-917513. doi: 10.1155/2015/917567.

Pan, X., Xiang, H., Ding, C., Liu Hua, Chen Guozhen, and Mao, T. (2012). Cost of two different therapies for end-stage renal disease in northwest China. *Journal of Medical Colleges of PLA（China）* 27(2)**,** 80-86. doi: 10.1016/S1000-1948(12)60009-4.

Rossini, M., Viapiana, O., Vitiello, M., Malavolta, N., La Montagna, G., Maddali Bongi, S., et al. (2017). Prevalence and incidence of osteoporotic fractures in patients on long-term glucocorticoid treatment for rheumatic diseases: the Glucocorticoid Induced OsTeoporosis TOol (GIOTTO) study. *Reumatismo* 69(1)**,** 30-39. doi: 10.4081/reumatismo.2017.922.

Rovin, B.H., Furie, R., Latinis, K., Looney, R.J., Fervenza, F.C., Sanchez-Guerrero, J., et al. (2012). Efficacy and safety of rituximab in patients with active proliferative lupus nephritis: The lupus nephritis assessment with rituximab study. *Arthritis Rheum* 64(4)**,** 1215-1226. doi: 10.1002/art.34359.

The Third Affiliated Hospital of Sun Yat-sen University (2019). Hospital Information System. Available from: <https://www.zssy.com.cn>.

Wang, J.J., Lu, Y.E., Shan-Lian, H.U., Hao, C.P., and Han, F. (2006). Disease burden on dialysis therapy. *Chinese Health Resources*.

Zhang, L., Lu, G.H., Ye, S., Wu, B., Shen, Y., and Li, T. (2017). Treatment adherence and disease burden of individuals with rheumatic diseases admitted as outpatients to a large rheumatology center in Shanghai, China. *Patient Prefer Adherence* 11**,** 1591-1601. doi: 10.2147/PPA.S144624.
